# Design of an Efficient PTB7:PC70BM-Based Polymer Solar Cell for 8% Efficiency

**DOI:** 10.3390/polym14050889

**Published:** 2022-02-23

**Authors:** Ahmed N. M. Alahmadi

**Affiliations:** Device Simulation Lab, Department of Electrical Engineering, Umm Al-Qura University, Makkah 21955, Saudi Arabia; anmahmadi@uqu.edu.sa

**Keywords:** polymer, solar cell, bulk heterojunction, PEDOT:PSS, PTB7:PC70BM, PFN-Br, SCAPS 1D

## Abstract

Polymer semiconductors may have the potential to fully replace silicon in next-generation solar cells because of their advantages such as cheap cost, lightweight, flexibility, and the ability to be processed for very large area applications. Despite these advantages, polymer solar cells are still facing a certain lack of power-conversion efficiency (PCE), which is essentially required for commercialization. Recently, bulk heterojunction of PTB7:PC70BM as an active layer showed remarkable performance for polymer solar cells in terms of PCE. Thus, in this paper, we developed and optimized a novel design using PEDOT:PSS and PFN-Br as electron and hole transport layers (ETL and HTL) for ITO/PEDOT:PSS/PT7B:PC70BM/PFN-Br/Ag as a polymer solar cell, with the help of simulation. The optimized solar cell has a short-circuit current (Isc) of 16.434 mA.cm^−2^, an open-circuit voltage (Voc) of 0.731 volts, and a fill-factor of 68.055%, resulting in a maximum PCE of slightly above 8%. The findings of this work may contribute to the advancement of efficient bulk-heterojunction-based polymer solar cells.

## 1. Introduction

Organic semiconductor-based solar cells have gained considerable popularity over the last few years, and some scientists believe they have the potential to completely replace silicon-based solar cells in the near future [1,2,3,4,5]. Organic semiconductors offer many advantages for solar cell applications such as lightweight, low cost, fabrication on various substrates, wide-area applications, and flexible and tunable processing at room temperature [6]. Despite these well-reported advantages, organic solar cell efficiency is far behind Si solar cells. It is generally believed that some combination of a proper absorber layer with a hole and electron may yield a high-efficiency device for next-generation solar cells [7,8,9,10].

Researchers are exploiting a variety of techniques to enhance the power-conversion efficiency (PCE) of organic solar cells. Some schools of thought still believe that the combination of the most suited hole, electron transport, and buffer layer with a highly efficient bulk-heterojunction as an absorber layer may yield an excellent photovoltaic response [11,12,13]. Bulk heterojunction has attracted great interest due to various advantages such as low cost, tunable bandgap and electron affinity, lightweight, and most importantly excellent power conversion efficiency compared to other organic/polymer materials. The bulk-heterojunction layer consists of a blend of acceptor and donor materials (organic/polymer) at the nanoscale and broadly speaking donor materials are usually polymer/organic while fullerene derivatives (PCBM) are used as acceptor materials for bulk heterojunctions layer such as P3HT:PCBM, MEH-PPV:PCBM, PCPDTBT:PCBM, and PTB7:PC70BM [14,15].

Suitable electron and hole transport layers (ETL and HTL) for PTB7:PC70BM create challenges, as PTB7:PC70BM has strong binding (low dielectric constant) energy for exciton with low diffusion length, and despite its heterogeneous nature most of the excitons are lost in recombination [11,12]. If a very thin PTB7:PC70BM layer is used, then these issues can be improved, but the issue of inefficient optical absorption will arise. On the other hand, the optimum thickness of the PTB7:PC70BM layer emphasizes the importance of an efficient hole and electron transport layer, which attract the required free carriers and also block the injection of opposite free carriers. The optical absorption spectra of PTB7:PCBM bulk-heterojunction polymer can be found in the reference [16].

For the hole transport layer, poly(3,4-ethenedioxythiophene):poly(styrenesulfonate) (PEDOT:PSS) is accepted as one of the best polymers for hole transport materials and especially for inverted polymer solar cells. It has many advantages such as lightweight, high conductivity, low cost, and thin-film processing even at room temperature [17,18]. However, the most important reason for its success as a hole transport layer is that PEDOT:PSS offers not only a well-coordinated work function for HOMO (Highest Occupied Molecular Orbital) level of the donor semiconducting polymer but also offers highly matched work function with ITO (tin-doped indium oxide) over a glass substrate [19]. As well as proper work function, PEDOTT:PSS also offers excellent visible transparency as well as good air stability essentially required for photovoltaic applications [20]. As a result, PEDOT:PSS can remove holes efficiently from the semiconducting polymer layer and forward them towards the cathode. Hence, in this work, we employed PEDOT:PSS as a HTL.

**Figure 1:**
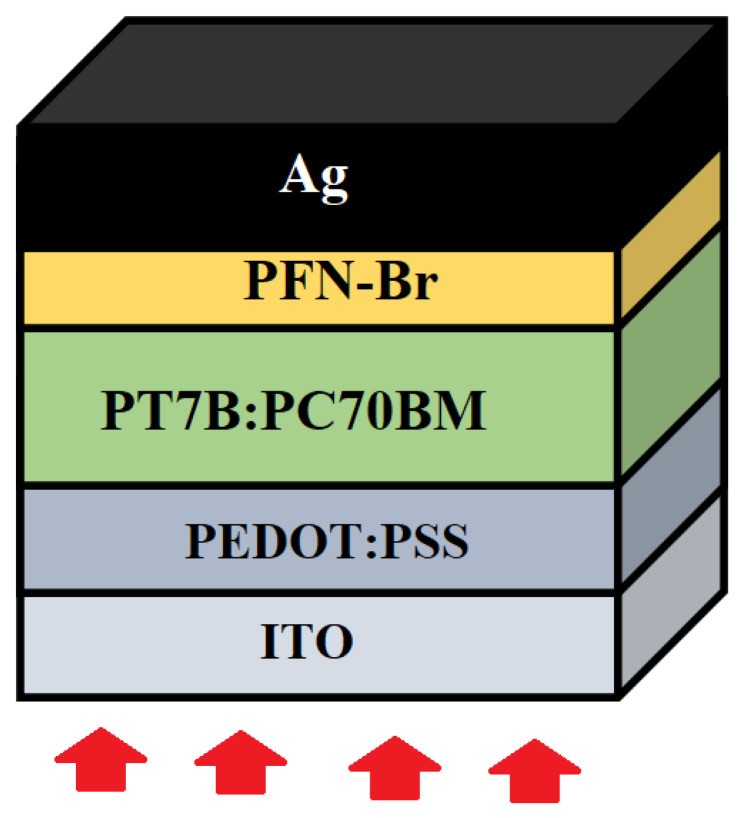
Shows the schematic view of the proposed ITO/PEDOT:PSS/PTB7:PC70BM/PFN-Br/Ag photovoltaic device for simulation.

Similarly, for an electron transport layer, [6,6]-phenyl C60 butyric acid methyl ester (PC60BM) is another common material for inverted (p-i-n) polymer solar cells. It facilitates the electron-transport process and has very high electron-affinity which helps to extract the electron efficiently [21]. However, it has some limitations which cause degradation to the PCE of polymer solar cells. Some of these limitations are low electron mobility, high leakage current, and recombination at interfaces [5]. On the other hand, a polyfluorene derivative such as PFN-Br is reported to show excellent electron extraction and transport behavior [22]. Figure 1 shows the overall architecture of the novel ITO/PEDOT:PSS/PTB7:PC70BM/PFN-Br/Ag photovoltaic device proposed for this study. The photovoltaic response of the solar cell described above was numerically simulated in order to identify the optimal doping density and thickness of ETL, HTL, and the absorber layer.

## 2. Simulation Methods and Physical Parameters

### 2.1. Simulation Software

Simulation of a photovoltaic response for an organic solar cell is a highly mature field and has already played a vital to overall improving the PCE of the solar cell. In industry, various types of software are available for the simulation of photovoltaic response. Among simulation software, SCAPS-1D is very attractive as open-source, simple, highly reliable, and provides comprehensive tools for simulations. Similarly, SCAPS-1D software also offers high consistency between simulation and experimental results [23,24,25]. On the other hand, various simulation results for organic/polymer materials as absorbers or transport layers for different solar cells have already been reported in the literature [26,27,28]. Therefore, SCAPS 1D software (SCAPS 3.8, ELIS-University of Gent, Gent, Belgium) was chosen for the simulation study of the proposed solar cell.

### 2.2. Simulation Method

SCAPS 1D simultaneously solves many fundamental semiconductor photovoltaic equations for both electron and hole separately such as (i) continuity equation, (ii) Poisson equations, (iii) charge transport equations, (iv) diffusivity equations, and (v) optical absorption equations. The following reference [29,30] contains in-depth information on these fundamental equations These equations are driven by physical and geometrical parameters associated with each layer and lead to the overall photovoltaic response of the given solar cell. These equations are listed as

(1) $$\frac{d^{2}\emptyset \left(x\right)}{dx^{2}}=\frac{q}{\in_{o}\in_{r}}\;\left(p\left(x\right)-n\left(x\right)+N_{D}-N_{A}+\rho_{p}-\rho_{n}\right)$$

(2) $$\frac{dJ_{n}}{dx}=G-R$$

(3) $$\frac{dJ_{p}}{dx}=G-R$$

(4) $$J=J_{n}+J_{p}$$

(5) $$J_{n}=D_{n}\;\frac{dn}{dx}+\mu_{n\;}n\frac{d\emptyset }{dx}$$

(6) $$J_{p}=D_{p}\;\frac{dp}{dx}+\mu_{p\;}p\frac{d\emptyset }{dx}$$

(7) $$\alpha \;(\lambda )=\left(A+\frac{B}{h\nu }\right)\;\sqrt{h\nu -E_{g}}$$

Here ∅(x), *q*, $\in_{o},\;\in_{r},\;\rho_{P},\;\;\rho_{N}$, *N_A_*, *N_D_*, *p*(*x*), *n*(*x*), *G*, *R*, *J_P_*, *J_n_*, and *J*, are the electrostatic potential, electrical charge, absolute permittivity of vacuum, relative permittivity of a semiconductor, hole defect density, electron defect density, shallow acceptor doping density, shallow donor doping density, hole carrier density as a function of the thickness (*x*), electron carrier density as a function of the thickness (*x*), carrier generation rate of free carriers, total carrier recombination rate, hole current density, and electron current density, total current density, respectively. Similarly, *D_p_*, *D_n_, µ_p_*, and *µ_n_* are the free hole diffusion coefficient, free electron diffusion coefficient, free hole carrier mobility, and free-electron carrier mobility, respectively. Finally, *h*, α (λ), Eg, and ν are the plank constant, absorption coefficient, energy bandgap, optical frequency, and few arbitrary constant, respectively.

**Figure 2:**
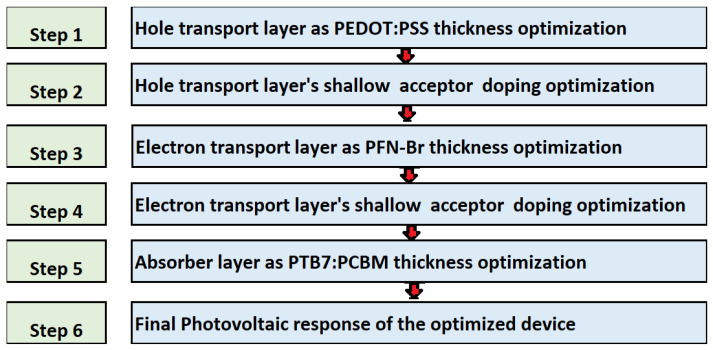
The steps of the methods followed in this work to optimize the proposed ITO/PEDOT:PSS/PTB7:PCBM/PFN-Br/Ag solar cell.

The simulation of the proposed solar cell is divided into six-well defined steps, these simulation steps are summarized as a flowchart in Figure 2. Firstly, the hole transport layers’ thickness and doping density are optimized and then the electron transport layers’ thickness and doping density are optimized. Similarly, in the next step, the absorber layer thickness is optimized, while in the second last and last step the final photovoltaic response of the optimized device is determined.

### 2.3. Physical Parameters

The physical parameters for each transport and absorber layer required by the software are the backbone of the simulation, special attention was paid to the selection of these parameters. These parameters are selected from the published results and are listed in Table 1. As organic semiconductor is considered disordered material, it inherently offers a high density of traps [31,32,33,34]. The photovoltaic performances of solar cells are seriously affected by the existence of both shallow and deep traps. Therefore, high traps density (10^15^ cm^−3^) is introduced in both bulk and layer interface for the hole/electron transport layer and absorber layer, as shown in Table 1. Similarly, all calculations were performed at an ambient temperature environment of 300 K with 100 mW/cm^2^ of power spectral density as a 1.5 AM solar radiation light source.

## 3. Results and Discussion

### 3.1. Thickness Optimization of PEDOT:PSS

**Figure 3:**
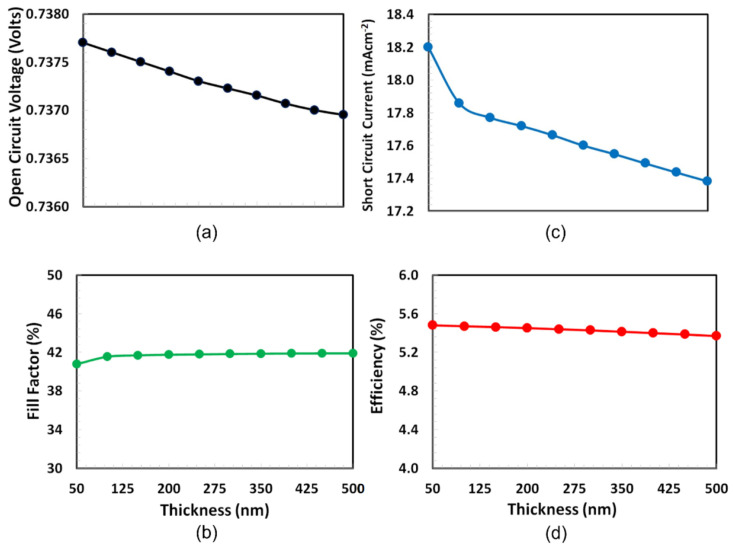
Performance characteristics such as (**a**) Voc, (**b**) fill factor, (**c**) Isc, and (**d**) PCE of the proposed ITO/PEDOT:PSS/PTb7:PC70BM/PFN-Br/Ag solar cell as a function of the PEDOT:PSS (HTL) thickness.

Thickness optimization of PEDOT:PSS as a hole transport layer is very crucial for the proposed solar cell because at one side PEDOT:PSS interacts with semitransparent ITO and on the other side it interacts with PT7B:PC70BM absorber layer. As a result, optical transmission, hole extraction and blocking of the electron from the absorber, hole transportation, and collection to the respective ITO anode depend critically on the PEDOT:PSS layer thickness [39]. The thickness optimization of PEDOT:PSS was performed by determining the photovoltaic characteristics such as PCE, short-circuit current (Isc), open-circuit voltage (Voc), and fill-factor, as functions of the thickness of PEDOT:PSS, shown in Figure 3. Among these photovoltaic parameters, fill-factor is unique and defined as the percentage ratio between the actual and maximum possible power.

The thickness range of PEDOT:PSS is selected from 50 nm to 500 nm according to their efficiency with the high repeatability for the photovoltaic response [40]. Figure 3 demonstrates that both open-circuit voltage and fill-factor, as well as short-circuit current and efficiency, follow different trends. At almost 125 nm, the fill factor of the cell hits a maximum and then nearly remains constant as the thickness of PEDOT:PSS increases, while Voc is sharply declined with the increase in PEDOT:PSS thickness. On the other hand, PCE and short-circuit current is dropped from 50 nm thickness of PEDOT:PSS. Because PCE is the decisive factor, the optimal thickness of PEDOT:PSS as an HTL for the current solar cell is 50 nm.

### 3.2. Shallow Doping Density Optimization of PEDOT:PSS

**Figure 4:**
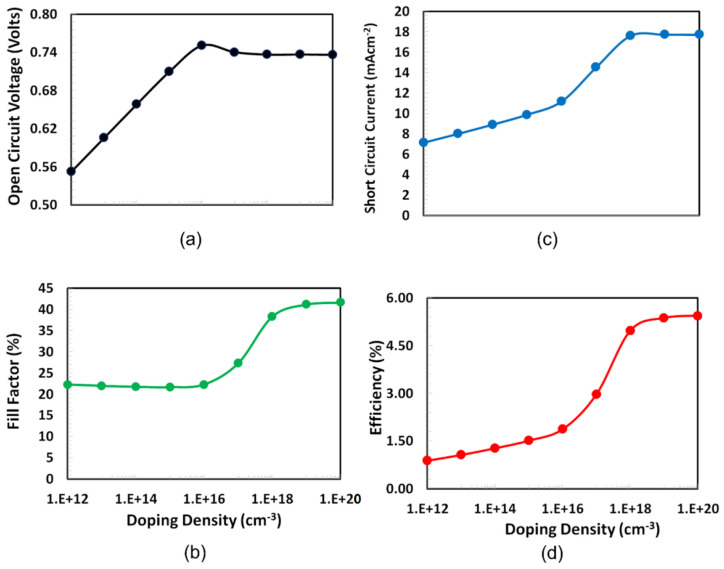
Performance characteristics such as (**a**) Voc, (**b**) fill factor, (**c**) Isc, and (**d**) PCE of the proposed ITO/PEDOT:PSS/PTb7:PC70BM/PFN-Br/Ag solar cell as a function of the PEDOT:PSS (HTL) doping density.

Another significant parameter to consider when optimizing a solar cell for efficiency is the doping density for PEDOT:PSS as the HTL. Doping of PEDOT:PSS as the hole transport layer significantly improves both charge extraction and charge transport process by reducing the series resistance and the establishment of ohmic contacts to the ITO electrodes, which overall enhances the solar cell’s photovoltaic parameters [41]. However, the higher dopant concentration may cause the creation of traps, which in turn behave as electron–hole recombination centers for PEDOT:PSS, thus we selected the range of doping density from 10^12^ to 10^20^ cm^−3^ based on published results [42]. PEDOT:PSS doping is critical for the proposed solar cell to have an efficient photovoltaic response. Before beginning the doping simulation, the optimized PEDOT:PSS thickness was updated in the software, and then photovoltaic parameters, such as Voc, fill factor, Isc, and PCE, as functions of shallow acceptor doping of PEDOT:PSS. The layer was simulated as shown in Figure 4. The figure depicts similar trends for all photovoltaic parameters except open-circuit voltage, which increases sharply and reaches a maximum at 10^16^ cm^−3^ and then starts to decrease. While other photovoltaic parameters also increase at early doping density with a slow rate, sharply rise to 10^18^ cm^−3^, and then slightly increase up to 10^20^ cm^−3^, which is the typical behavior of trapped space charge, limited current behavior was also observed for many organic/polymer semiconductors [43,44,45]. Consequently, the optimal doping density for the PEDOTPSS (HTL) in the proposed solar cell is inferred to be 10^20^ cm^−3^.

### 3.3. Electron Transport Layer Thickness Optimization

**Figure 5:**
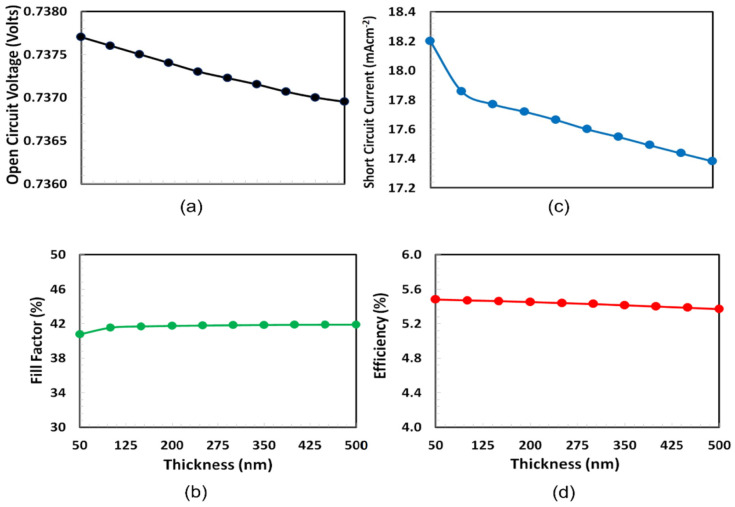
Performance characteristics such as (**a**) Voc, (**b**) fill factor, (**c**) Isc, and (**d**) PCE of the proposed ITO/PEDOT:PSS/PTb7:PC70BM/PFN-Br/Ag solar cell as a function of PFN-Br (ETL) thickness.

The optimal thickness of PFN-Br as an ETL is obtained in the third step of the simulation. Just like PEDOT:PSS as the HTL, the thickness of PFN-Br as ETL is also very important for electron extraction from PT7B-PC70BM, electron transport, and collection of electrons at the Ag cathode. The thickness optimization of PFN-Br was performed by determining the photovoltaic characteristics such as Voc, fill factor, Isc, and PCE as functions of PFN-Br thickness, shown in Figure 5. The Voc and Isc response of the current solar cell are degraded when the thickness of PFN-Br increases, while fill-factor is slightly increased up to 125 nm and then remains nearly constant. The efficiency is also degraded but at a very slow rate. The figures clearly show that the optimal thickness of PFN-Br as an ETL is 50 nm. Therefore, it can be inferred that the 50 nm thickness of PFN-Br provides the balance trade-off between electron–hole recombination, electron extraction, and blocking of the hole from the absorber, electron transportation, and hence collection to the respective Ag cathode. 

### 3.4. Shallow Doping Density Optimization of the PFN-Br

**Figure 6:**
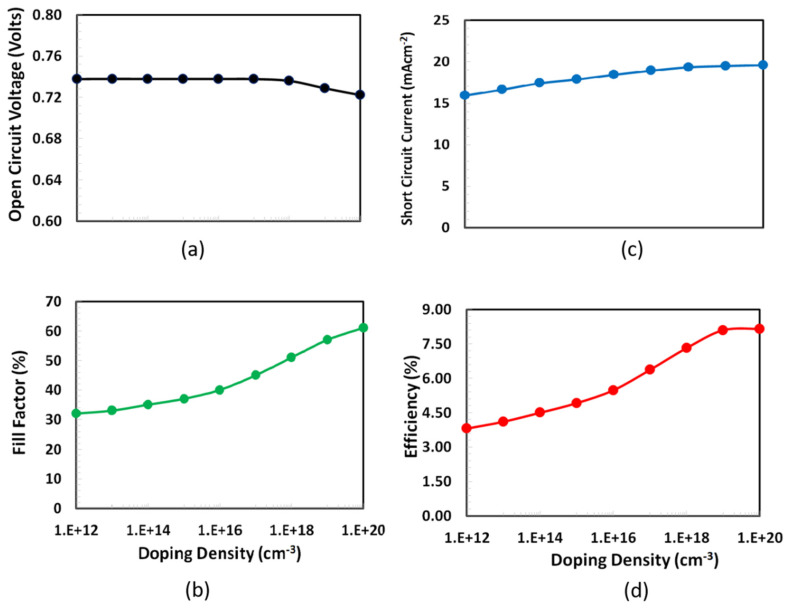
Performance characteristics such as (**a**) Voc, (**b**) fill factor, (**c**) Isc, and (**d**) PCE of the proposed ITO/PEDOT:PSS/PTb7:PC70BM/PFN-Br/Ag solar cell as a function of PFN-Br (electron-transport layer) doping density.

In the fourth step of the simulation, the optimum doping density of PFN-Br as an electron transport layer is determined. The optimized donor doping of PFN-Br can be attributed to the efficient electron extraction and good ohmic contact between Ag cathode and the active PTB7:PC70BM layer. The optimized donor doping PFN-Br was estimated by determining the photovoltaic parameters, such as Voc, fill factor, Isc, and PCE, by altering the shallow donor doping of PFN-Br from 10^12^ to 10^20^ cm^−3^, as shown in Figure 6. According to the Figure, it can be seen that higher doping of PFN-Br causes the open-circuit voltage response to degrade, which may be due to the creation of extra traps density at higher doping and the relaxation of the free carriers at these traps may cause to reduce the open-circuit voltage [46]. While PCE, fill-factor, and short-circuit current are increased with doping, PCE performed well, reaching the maximum at 10^18^ cm^−3^ doping and then starting to degrade. Thus, on the basis of these results, it can be concluded that the most optimal doping for PFN-Br as an ETL is 10^18^ cm^−3^.

### 3.5. Thickness Optimization of PTB7-PC70BM

**Figure 7:**
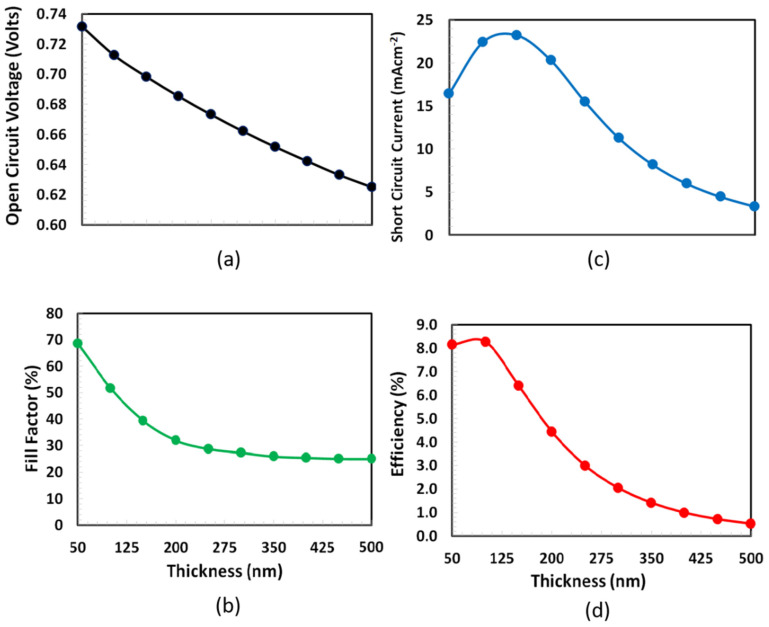
Performance characteristics such as (**a**) Voc, (**b**) fill factor, (**c**) Isc and (**d**) PCE of the proposed ITO/PEDOT:PSS/PTb7:PC70BM/PFN-Br/Ag solar cell as a function of PTB7:PC70BM (absorber layer) thickness.

Thickness optimization of bulk-heterojunction polymer absorber layer (e.g., PTB7-PC70BM) is one of the main challenging tasks because it depends on many inter-related processes such as strong optical absorption, generation of electron–hole pairs, conversion of bounded electron–hole pairs into free carriers, reducing carrier recombination losses, efficient charge transportation to the respective transport layers, mechanical and environmental stability. All these factors required different thicknesses of the absorber layer for their efficient individual response and a compromise between these processes is required for an efficient photovoltaic response [47,48,49]. In literature, various thicknesses of bulk heterojunction absorber layer for organic/polymer solar cells are reported [50,51,52]. Therefore, we varied the thickness of PTB7:PC70BM from 50 to 500 nm for simulation. Consequently, the thickness optimization of bulk heterojunction PTB7:PC70BM absorber layer was performed by simulating the photovoltaic characteristics such as Voc, fill factor, Isc, and PCE by altering the thickness of absorber layer and the results are shown in Figure 7. Both Voc and fill-factor decrease with thickness, while PCE and short-circuit current, initially, slightly increase up and reached the maximum at nearly 100 nm thickness, then they gradually decrease. Hence, based on the simulation results, it can justify that the 100 nm thickness of the PTB7:PC70BM is the optimum thickness of the current solar cell.

### 3.6. Photo Current–Voltage Response of Proposed Solar Cell

**Figure 8:**
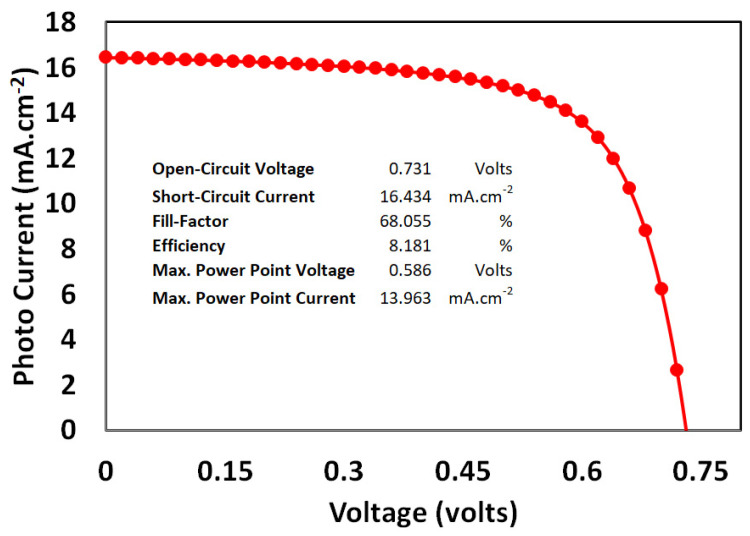
The simulated photocurrent–voltage response at AM 1.5 for the proposed ITO/PEDOT:PSS/PT7:PC70BM/PFN-Br/Ag solar cell.

The final phase of the simulation was to combine all of the optimum doping density and thickness for the PEDOT:PSS, PFB-Br, and PTB7:PC70BM layers and determine the current solar cell’s overall photocurrent–voltage response, as shown in Figure 8.

The proposed solar cell’s photovoltaic parameters are shown in Figure 8. The optimized ITO/PEDOT:PSS/PTB7:PC70BM/PFN-Br/Ag solar cell has an Isc of 16.434 mA.cm^−2^, Voc of 0.731 volts, a fill-factor of 68.055%, and a PCE of 8.18%. The higher value of short-circuit current may be due to the commutative effects of wider optical absorption, exciton generation, efficient exciton dissociation leads to the free carrier generation, and then transportation at their respective transport layer before collection at electrodes [53]. The proposed solar cell’s open-circuit voltage still has space for future improvement.

On the other hand, lower PCE compared to the other reported simulation of bulk-hybrid solar cells is maybe due to the incorporation of a higher density of traps [38]. It is experimentally evident that polymers are full of traps, and these traps may be presented due to many factors such as humidity, structural defects, distortion, impurity, and/or any other known or unknown reasons. However, these traps act as the recombination centers and cause severely degrade the overall photovoltaic response. Therefore, a high density of traps in each layer is introduced in order to make the simulation more realistic and comparable to the experimental results, which in turn show the lower PCE.

## 4. Conclusions

In conclusion, we have efficiently designed and optimized a polymer-based novel bulk heterojunction solar cell as ITO/PEDOT:PSS/PTB7:PC70BM/PFN-Br/Ag through SCAPS 1D simulations. For this purpose PEDOT:PSS, PFN-Br, and PT7B:PC70BM layers were selected as a HTL, ETL, and bulk-heterojunction absorber layers, respectively, and sandwiched between transparent ITO and Ag electrodes. Doping density and thickness of both PEDOT:PSS and PFN-Br were optimized and then PT7B:PC70BM is investigated for an efficient photovoltaic response. The proposed ITO/PEDOT:PSS/PTB7:PC70BM/PFN-Br/Ag solar cells yield an Isc of 16.434 mA.cm-2, a Voc of 0.731 volts, and a fill factor of 68.055%, resulting in a PCE of just over 8 %. Similarly, it is also indicated that all photovoltaic parameters are considerably affected by the doping density as well as the layer thickness of both ETL and HTL, and the bulk-heterojunction absorber layer. The higher short circuit current may the result of efficient optical absorption, exciton generation, exciton dissociation, free carrier generation, and then transportation at their respective transport layers before collection at the electrodes. As it is accepted that polymers are full of traps, we introduced a high density of traps in each layer in order to make the simulation more realistic, which in turn shows the lower PCE. Additionally, the proposed solar cell’s open-circuit voltage still has room for improvement.

## Figures and Tables

**Table 1:** The parameters of the photovoltaic device utilized in these simulations, including the initial estimation of the doping concentrations and thicknesses of each layer, which will be improved in the subsequent stages.

Physical Parameters	Symbol	Unit	PEDOT:PSS	PTB7:PC_70_BM	PFN-Br
Thickness	Th	Nm	-	250	250
Energy Band Gap	E_g_	eV	1.6	0.9	2.98
Electron Affinity	Χ	eV	3.5	3.7	4
Dielectric Permittivity (Relative)	Ε	-	3	3.9	5
Effective Density of States at Valence Band	N_V_	cm^−3^	1 × 10^22^	1 × 10^18^	1 × 10^19^
Effective Density of States at Conduction Band	N_C_	cm^−3^	1 × 10^22^	1 × 10^18^	1 × 10^19^
Hole Thermal Velocity	V_e_	cm/s	1 × 10^7^	1 × 10^7^	1 × 10^7^
electron Thermal Velocity	V_h_	cm/s	1 × 10^7^	1 × 10^7^	1 × 10^7^
Electron Mobility	μ_e_	cm^2^/V.s	0.01	5.00 × 10^−4^	1.00 × 10^−4^
Hole Mobility	μ_h_	cm^2^/V.s	9.9 × 10^−0.5^	5.00 × 10^−4^	2.00 × 10^−6^
Uniform Shallow Donor Doping	N_d_	cm^−3^	0.00	1 × 10^19^	-
Uniform Shallow Acceptor Doping	N_a_	cm^−3^	-	1 × 10^19^	0
Defect Density	N_t_	cm^−3^	1 × 10^15^	1 × 10^15^	1 × 10^15^
References			[35]	[36,37]	[38]

## Data Availability

Not applicable.
